# Treatment of Abscess

**Published:** 1897-09

**Authors:** 

**Affiliations:** Flint


### Treatment of Abscess.

BY DR. MONROE, OF FLINT.

Read before the Michigan State Dental Society.

It is not so many months ago that I have ceased to remember with pleasure the last dental convention I attended.

I recall to mind with a great deal of satisfaction the benefits which I received, in the way of personal experiences, related by members of that convention.

From the narration of more than one of these experiences, I received suggestions which are of great benefit to me in my every-day practice.

It has been my fortune to have some experience in a certain line of work, which is attracting no little attention at present in our dental journals. While I cannot hope that I am telling you any thing new, I must at least hope to succeed in interesting you to a limited extent.

Two years ago a gentleman about forty-five years of age came into my office and said, "I have some teeth that trouble me. They have been troubling me for over a year. I have been in two offices during that time and they had started to treat them, but it hurt so badly that I never went back to either of them for a second treatment. I don't want to be hurt, I would prefer the toothache rather than to suffer pain in a dental chair."

Most of his posterior teeth had been extracted under an anaesthetic, and he was depending largely on his anterior ones for mastication. The troublesome tooth was the left cuspid superior, which was slightly raised in its socket, and a little loose. The lateral and central incisors next to it were also devitalized.

Knowing that I would probably not be able to get him in the chair for a second treatment, I determined to try to make it a clean operation at one sitting.

I opened all three teeth through their root canals, wiped out all the pus I could press out through the canal of the cuspid, washed it out carefully with warm, carbolized water, forced carbolic acid and iodine, equal parts, into the pus cavity and filled it with chloro-percha and gutta percha points. I then cleaned out the canals of the central and lateral, sterilized them, and filled them in the same way. I told him that they would probably trouble him for a day or so, but after that they might never bother him.

I met him on the street in a few days and he said that the night after the treatment he slept little or not at all. The gum had swollen and burned, as he explained it, but they were then all right. In about two months from that time, I put in permanent fillings and they have not troubled him since. I had occasion to treat the lingual root of a superior right second molar, which would have been almost impossible to open through the palatal side, in the same manner as the case above mentioned, with the same results, a little more than a year ago. Yet, on account of the trouble following, I have never followed this for a practice and, in fact, have extracted molars in preference.

Prior to and since that time I have treated similarly affected teeth by cleaning out the root canals, through the apex of the root by using Gates' glidden drills, if necessary, or even a fifty percent solution of sulphuric acid. Then, after injecting about a one and one-half percent solution of cocaine to a mixture of tr. iodine, carbolic acid, sulph. morphia, atropine sulph. (pot., iod.), glyc., and water into the gum, over the apex of the root, which renders the gum entirely painless, lance the gum in the form of a cross, and with a large, round, sharp bur, drill through the process. Completing this work with a sharp fissure bur, with which it is an easier matter to cut off the end of the root or drill out any diseased bone in the pus cavity. The small particles of process and tooth are now washed out, first with warm, carbolized water, then with peroxide of hydrogen, followed by carbolic acid and iodine, equal parts, forced through the canal and out through the fistula, leaving a thread of cotton saturated in carbolic acid and iodine in the canal, with another similar one in the fistula. The after-treatment consisted in simply washing out the cavities with peroxide of hydrogen, unless the pus continued to form, when the first operation would be repeated. Sometimes a cure would be brought about after the first treatment, and again, after two or three months' treatment, the tooth would have to be extracted.

At the time of our last convention in Grand Rapids, I had a right superior central incisor, that I had been treating in this way for about two months, with no hope of success. I consulted several of the doctors present in regard to it and received as many different replies. As they all had some failures, I concluded that I would have to extract it. But before doing so, I cleaned it out thoroughly, sterilized it well through the canal and fistula and filled the canal permanently, after I had thoroughly dried it with tannic acid and cotton. The patient remarked that the tooth felt better than it had at any previous treatment. Every few days I would wash out the fistula, which remained open of itself, due to the purulent discharge that had no other avenue of escape, with warm, carbolized water, and inject a few drops of tr. iodine into the pus cavity. In a short time the opening healed up and has since caused no trouble.

Since that time I have been filling the root canals of abscessed teeth at the first sitting, after the opening has been made through the process and the pus cavity thoroughly burred out, and cleansed. I fail to see of what benefit it is to leave the root and canal unfilled. To my mind, it would be as easy to treat a chronic case of appendicitus through the oral cavity, as to treat a chronic, abscessed tooth through the root canal. When we thoroughly clean out a root canal, open well through the apex to the fistula, we have in my opinion, done all we can through that channel. It is better to fill it at once, and the fistula will allow any pus to escape that might otherwise be inclosed. Should any pus form afterward, it is evident that we have a diseased tooth to drill out, which can be reached only through the fistulous opening. That we have a diseased tooth and not process, is proven by the fact, that when we extract an abscessed tooth, the cavity left, generally heals up in a few days, causing no further trouble. After cutting off the end of the root, we have the diseased portion extracted, and the cavity, represented by the fistula, equals the cavity made, were the tooth was extracted.

This is the way I, with my limited experience, view the matter and if my views are mistaken or exaggerated, my heartfelt thanks will be given to him who shows me wherein I have erred.

DISCUSSION.

Dr. Williams: I would like to know if it would not be of more benefit to leave the canal open instead of filling it permanently. I fill it with cotton which is not so difficult to extract as gutta percha.

Dr. Monroe: I do not think it necessary to remove the filling. The difficulty would be at the root or in the process. I think a tooth can be over-treated. The point I wished to make, was, that of filling the tooth at the first sitting.

Dr. House: I would like to ask Dr. Monroe if that pertains to teeth where the pulp has become devitalized, where it has decomposed, where the pulp is dead, or where it is absorbed entirely. We often find teeth where the pulp is devitalized and the flow of pus difficult to arrest. Also cases where the pulp is absorbed and we have a dry condition to act upon.

Dr. Monroe: I had a case yesterday of that kind, which I am to extract. I should treat it first, for the reason that it is sore. It is a left lateral incisor and there is a hard lump under the eye. I believe in that case it would be better to wait. It would be easier for the patient. But in the case of the tooth being either dry, or pus coming through the root canal, clean it out thoroughly and sterilize, if blood or serum continues through the canal, use tannin and cotton. Sometimes it will take an hour and a half to dry and fill it. In the case to which I referred, the patient said the tooth felt all right. The fistula is still open.

Dr. Warboys: I would like to ask Dr. Monroe if he has ever had a recurrence of an abscess after that kind of treatment after he considered it entirely healed up?

Dr. Monroe: More often than otherwise. Even after cutting off a portion of the root.

Dr. Young: I have always considered it best to fill the tooth before cutting off the root, because after cutting it off you must have a larger opening, it is more difficult to fill on account of pushing the filling farther than you wish it to go. I think a permanent filling helps to force the pus out.

Dr. Morgan: I have claimed for a long time that it was impossible to make a lower molar healthy after once diseased. If the fistula does not return for four or five months, we have been successful. I have met with better success, if after opening into the root, I had used a hot instrument at once before washing. Most of the discharge would come away with the hot instrument. I have always been very careful not to drill through the end of the root. If an upper front tooth usually the opening at the end of the root is large enough to force the medicine through without drilling.

Dr. Monroe: I have found oftentimes that by drilling through the tooth, getting a circuit through the fistulus opening and root canal and after drying thoroughly and filling, that the fistula would heal itself.

